# Combined Endocardial Radiofrequency and Pulsed-Field Left Ventricular Summit Ablation: With Great Power Comes Great Responsibility

**DOI:** 10.1016/j.jaccas.2025.106304

**Published:** 2025-12-17

**Authors:** Gustavo S. Guandalini

**Affiliations:** Cardiac Electrophysiology Section, Division of Cardiovascular Medicine, Hospital of the University of Pennsylvania, Philadelphia, Pennsylvania, USA

**Keywords:** left ventricular summit, premature ventricular contraction, pulsed-field ablation, radiofrequency ablation

**Figure:**
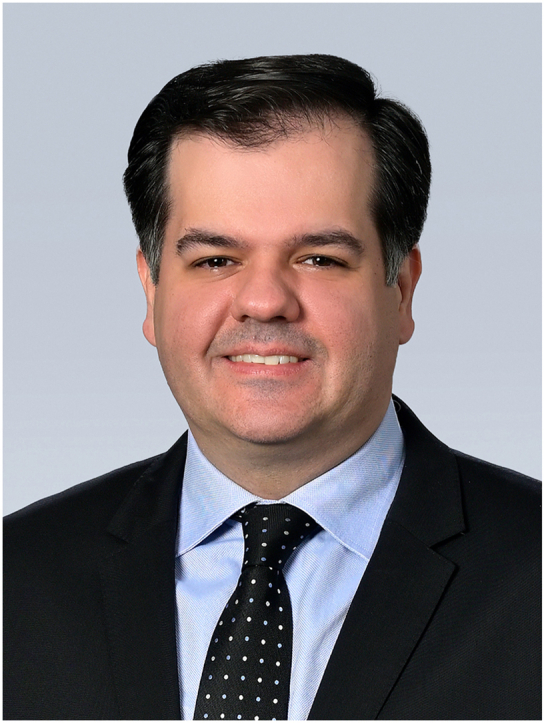

Premature ventricular contraction (PVC) ablation from the left ventricular outflow tract (LVOT) has been considered more challenging than that from the right ventricular outflow tract (RVOT), owing not only to increased procedural risk from catheter manipulation within the arterial circulation but also to decreased procedural success from increased myocardial thickness and anatomic complexity. This is acknowledged in the expert consensus on catheter ablation of ventricular arrhythmias: RVOT PVC ablation carries a class of recommendation I indication as the first-line therapy, in contrast to class of recommendation IIa for LVOT PVCs (when antiarrhythmics are ineffective, not tolerated or not desired).^1^ In experienced centers, LVOT PVC ablation is often performed as first-line therapy with excellent outcomes, but those arising from the left ventricular (LV) summit remain significantly challenging.

As defined by McAlpine, the LV summit describes the uppermost point of the LV epicardium, where the upper end of the anterior interventricular sulcus joins the aortic portion of the LV ostium.^2^ This epicardial anatomic location is often invoked as the site of origin when outflow tract PVC ablation fails from endocardial RVOT and LVOT (including the right and left sinuses of Valsalva), attributable to insufficient tissue penetration with traditional radiofrequency (RF) ablation. In practical terms, a PVC can be considered to originate from the LV summit when epicardial activation mapping (either from the anterior interventricular vein [AIV] or direct epicardial mapping via percutaneous approach) demonstrates earliest activation compared to neighboring structures. Nevertheless, catheter ablation from these mapping sites remains quite challenging. Ablation from the AIV can be limited by difficulty advancing the catheter beyond the great cardiac vein; when this is accomplished, high impedance can limit RF current delivery, and proximity to the left anterior descending artery poses risk of myocardial infarction. Likewise, direct LV summit ablation from the pericardial space not only carries the usual risks from percutaneous epicardial access but is also limited by proximity to epicardial coronary arteries and annular epicardial fat that prevents RF current delivery.^3^

Several strategies have been tried to increase endocardial ablation success for epicardial LV summit arrhythmias. These include anatomical ablation from endocardial vantage points (either RVOT or LVOT) near the epicardial site of origin^4^^,^^5^ and prolonged-duration endocardial ablation.^6^ More recently, systematic intramyocardial mapping via septal perforator veins revealed intramural origin in a significant proportion of these patients, differentiating the superior intraseptal space from the true epicardial LV summit and explaining occasional successful endocardial ablation despite the lack of presystolic activation.^7^

In this issue of *JACC: Case Reports*, Prasitlumkum et al^8^ describe a promising novel strategy for endocardial LV summit PVC ablation. Durable success is eventually achieved with pulsed-field ablation (PFA) after preconditioning with RF application from the endocardial LVOT despite barely presystolic activation. Such off-label use of a dual-energy ablation catheter was justified after failing most of the strategies previously described to increase procedural success, including ablation from adjacent RVOT and LVOT and epicardial ablation from direct percutaneous access and from the AIV. A holy grail in ventricular arrhythmia ablation, safe transmural lesion delivery has been previously tried with lower ionic strength irrigants, simultaneous unipolar and bipolar ablation, retractable needle-tip electrode catheter, and alcohol ablation.^9^ This report showcases sequential RF and PFA dual-energy delivery emerging as a viable alternative to achieve transmural ablation in the left ventricle.

A word of caution, however, is necessary before this becomes widely adopted. First, intramural mapping was not performed, so the reason for success could have been superior intraseptal space site of origin (thus more accessible with endocardial ablation). Still, the very presystolic activation from the AIV despite rS local electrogram strongly supports a true epicardial site of origin with full-thickness ablation achieved. Second, this was utilized as a bailout after extensive mapping of all neighboring structures. Therefore, despite the lack of significant presystolic activation, there was close anatomic proximity to the true site of origin before delivering sequential RF and PFA. Finally, the ability to achieve transmural myocardial ablation raises concerns over long-term effects to the ablated myocardium—including risk of acute or late rupture—and adjacent coronary arteries—including acute coronary vasospasm or late coronary injury. The authors are to be congratulated on using this great power with great responsibility, acknowledging its unknown effects while appropriately employing it in a very justified clinical setting.

## Funding Support and Author Disclosures

This work was supported by the Mark Marchlinski EP Research & Education Fund. The authors have reported that they have no relationships relevant to the contents of this paper to disclose.
